# Dermatose flagellée induite par bléomycine: un nouveau cas Clinique

**DOI:** 10.11604/pamj.2016.25.86.9784

**Published:** 2016-10-17

**Authors:** Salmi Narimane, Hassan Errihani

**Affiliations:** 1 Service d’Oncologie Médicale, Institut National d’Oncologie, Rabat, Maroc

**Keywords:** Erythème flagellé, BEP, bleomycine, Flagellate erythema, BEP, bleomycin

## Image en médecine

Nous rapportons le cas d’une patiente âgée de 56 ans, suivie dans notre institut pour une tumeur de granulosa de l’ovaire ayant reçu une chirurgie complète suivie d’une chimiothérapie adjuvante selon le protocole BEP (Bléomycine dose totale de 30 mg à J1, J8 et J15, Etoposide 100mg/m^2^ de J1 à J5, Cisplatine 20mg/m^2^ de J1 à J5). Après sa 2^ème^ cure de BEP, la patiente a présenté plusieurs hyperpigmentations linéaires cutanées au niveau de l’étage supérieur des régions thoraciques antérieure et postérieure ainsi qu’au niveau du bas du dos, sans notion de prurit, correspondant à l’érythème flagellé induit par la Bleomycine. Ces lésions cutanées sont apparues malgré l’utilisation d’une prémédication par antihistaminique et ont persisté après arrêt du traitement. L’érythème flagellé appelé, aussi dermatose flagellée, complique un traitement par Bleomycine dans 20% à 30% des cas. Généralement, cette toxicité survient après une dose cumulative de 90 à 285 mg, mais certains cas ont été reportés à partir des doses de 15 mg. C’est une pathologie bénigne dont l’apparition ne doit pas compromettre la poursuite de la Bleomycine.

**Figure 1:**
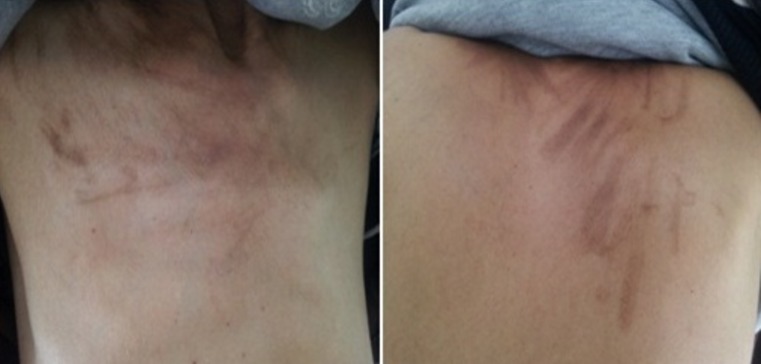
Pigmentations linéaires cutanées correspondant à une dermatose flagellée induite par la bleomycine

